# A critical assessment of estimating census population size from genetic population size (or vice versa) in three fishes

**DOI:** 10.1111/eva.12496

**Published:** 2017-07-04

**Authors:** Matthew Carl Yates, Thais A. Bernos, Dylan J. Fraser

**Affiliations:** ^1^ Department of Biology Concordia University Montreal QC Canada; ^2^ Group for Interuniversity Research in Limnology and Aquatic Environments (GRIL) Universite du Quebec Trois‐Rivieres QC Canada

**Keywords:** conservation biology, conservation genetics, effective population size, fisheries management, inventory and monitoring, wildlife management

## Abstract

Technological and methodological advances have facilitated the use of genetic data to infer census population size (N_c_) in natural populations, particularly where traditional mark‐and‐recapture is challenging. The effective number of breeders (N_b_) describes how many adults effectively contribute to a cohort and is often correlated with N_c_. Predicting N_c_ from N_b_ or vice versa in species with overlapping generations has important implications for conservation by permitting (i) estimation of the more difficult to quantify variable and (ii) inferences of N_b_/N_c_ relationships in related species lacking data. We quantitatively synthesized N_b_/N_c_ relationships in three salmonid fishes where sufficient data have recently accumulated. Mixed‐effects models were analysed in which each variable was included as a dependent variable or predictor term (N_b_ from N_c_ and vice versa). Species‐dependent N_b_/N_c_ slope estimates were significantly positive in two of three species. Variation in species slopes was likely due to varying life histories and reinforce caution when inferring N_b_/N_c_ from taxonomically related species. Models provided maximum probable estimates for N_b_ and N_c_ for two species. However, study, population and year effects explained substantial amounts of variation (39%–57%). Consequently, prediction intervals were wide and included or were close to zero for all population sizes and species; model predictive utility was limited. Cost‐benefit trade‐offs when estimating N_b_ and/or N_c_ were also discussed using a real‐world system example. Our findings based on salmonids suggest that no short cuts currently exist when estimating population size and researchers should focus on quantifying the variable of interest or be aware of caveats when inferring the desired variable because of cost or logistics. We caution that the salmonid species examined share life‐history traits that may obscure relationships between N_b_ and N_c_. Sufficient data on other taxa were unavailable; additional research examining N_b_/N_c_ relationships in species with potentially relevant life‐history trait differences (e.g., differing survival curves) is needed.

## 1 INTRODUCTION

Rapid technological and methodological advances in molecular genetics have increased interest in using genetic data to estimate or infer census population size (N_c_), especially where counting individuals is challenging (e.g., in large populations, elusive species or extremely remote locations) (Baldigo, Sporn, George, & Ball, 2017; Fraser, Calvert, Bernatchez, & Coon, 2013; Guschanski et al., 2009; Luikart, Ryman, Tallmon, Schwartz, & Allendorf, 2010; Ovenden et al., 2016). While direct individual counts could be obtained from comprehensive genetic surveys (e.g., Guschanski et al., 2009), methodologies that indirectly estimate N_c_ from environmental DNA (eDNA) or subsamples of individuals from a population represent potentially cost‐effective means through which census sizes could be estimated. Although eDNA is emerging as a potential method through which N_c_ could be inferred (Baldigo et al., 2017; Lacoursière‐Roussel, Côté, Leclerc, Bernatchez, & Cadotte, 2016), its application for this purpose remains relatively novel. In comparison, the scientific literature examining methodologies for estimating the contemporary effective population size (N_e_) of natural populations is relatively well developed.

The effective size of a population is a central evolutionary parameter influencing the extent of genetic drift, inbreeding and response to natural selection in isolated populations. Contemporary N_e_ (as opposed to long‐term N_e_, see Wang, 2005) represents a potentially useful tool to infer N_c_ because it can be linked specifically to recent cohorts and can be estimated from a (relatively) small number of genetic samples collected during a single collection event or over multiple temporal periods (Palstra & Fraser, 2012; Waples & Do, 2008). Understanding the conditions under which contemporary N_e_ (or its analogues) and N_c_ are associated with each other is highly valuable for conservation: it may be possible to use N_e_ to predict or monitor N_c_ (or vice versa) provided that relationships between N_e_ and N_c_ exist among or within populations and/or taxonomic groups (Bernos & Fraser, 2016; Ovenden et al., 2016; Tallmon et al., 2010; Whiteley et al., 2015).

For species with overlapping generations, the comparison of genetic and census population size can be made by comparing N_c_ to how many of those adults effectively contribute their genes to a single cohort, termed the effective number of breeders (N_b_) (it should be noted, however, that this is dependent on the capacity to assign individuals to specific cohorts) (Waples & Do, 2010). With minimal life‐history information, N_b_ can be used to infer contemporary N_e_ (Waples, Luikart, Faulkner, & Tallmon, 2013) and substitute for N_e_ when attempting to predict N_c_. N_b_ also provides valuable insights into the eco‐evolutionary dynamics of a population because interannual variation in N_b_ may be attributable to differences in individual adult reproductive success, family survival, and the overall number of families comprising the cohort (Waples & Antao, 2014; Whiteley et al., 2015).

Several recent studies have estimated N_b_ and N_c_ within multiple populations of the same species (e.g., Beebee, 2009; Bernos & Fraser, 2016; Ferchaud et al., 2016; Hoehn, Gruber, Sarre, Lange, & Henle, 2012; Perrier, April, Cote, Bernatchez, & Dionne, 2016; Whiteley et al., 2015). They identified important biological factors shaping N_b_/N_c_ within species, such as habitat limitations, life‐history traits or density dependence (Belmar‐Lucero et al., 2012; Bernos & Fraser, 2016; Whiteley et al., 2013). Time series of N_b_ and N_c_ revealed that the two variables were positively correlated but that N_b_/N_c_ was variable among populations and across years. Those results provided mixed support for the usefulness of one variable to infer the other in a monitoring context (Bernos & Fraser, 2016; Ferchaud et al., 2016; Whiteley et al., 2015). By comparison, few empirical investigations of the relationship between N_b_ and N_c_ among species have been conducted (Gomez‐Uchida, Palstra, Knight, & Ruzzante, 2013; Osborne, Davenport, Hoagstrom, & Turner, 2010). Such comparisons would be extremely practical for determining how concordant N_b_/N_c_ ratios are among taxonomically related species, an especially pertinent issue for rare species that lack data. It would also allow researchers to better understand factors contributing to variation in N_b_/N_c_ in natural populations with contrasting biology or life history.

Most taxa still have little N_b_/N_c_ data emerging, but sufficient data have become recently available in three fishes from the *Salmoninae* subfamily (Chinook salmon, Atlantic salmon and brook trout); these species form the basis of our quantitative analysis of N_b_/N_c_ relationships. The studies examining these species have largely found positive relationships between N_b_ and N_c_ (e.g., Bernos & Fraser, 2016; Ferchaud et al., 2016; Perrier et al., 2016; Van Doornik, Waples, Baird, Moran, & Berntson, 2011; etc.); we collated data across studies to produce models for converting N_b_ to N_c_ (and vice versa) in each species. We then evaluated the efficacy of using one population variable to infer the other by generating population size parameter prediction intervals under which novel previously unsampled populations with only a single known population size variable (either N_b_ or N_c_) are likely to fall. We also explored whether N_b_/N_c_ curves differed for taxonomically related species and whether they could be used to infer population size parameters across species. Lastly, the monetary cost‐benefit trade‐offs of estimating N_b_ or N_c_ are discussed using a real‐world example system in which N_b_/N_c_ estimates were obtained across twelve populations.

## 2 MATERIALS AND METHODS

### 2.1 Primary Literature review

To find articles in which both N_b_ and N_c_ estimates were obtained for the same populations, keyword searches were conducted on the academic search engine ISI Web of Science^™^. A complete keyword search for “Effective number of breeders” was performed. Relevant references within retrieved studies were also searched for usable data. The goal of the analysis was to derive linear relationships between N_b_ and N_c_ across multiple species. A particular species was therefore only included in the final data set if a minimum of 10 total N_b_/N_c_ estimates from at least three different populations were found; the only taxa with species for which these data requirement were satisfied were salmonids. A subsequent search of “Effective population size salmon*” was subsequently conducted to find any additional salmonid references missed by the initial search; this search found a single additional article. In most cases, N_c_ was “correctly linked” with brood year; that is, each N_b_ estimate generated from a cohort was matched with the N_c_ estimate of the parental generation. Only in two cases were N_b_ and N_c_ incorrectly linked; these were estimates taken from populations in which N_c_ and N_b_ were estimated for the same year (i.e., researchers only sampled the population once). Both estimates were still included in the final data set.

Multiple methods exist to estimate N_b_ which make use of either linkage disequilibrium, heterozygote excess, molecular coancestry or sibship frequency information obtained from genetic markers (Wang, 2016). Although there is currently debate regarding which estimators perform best under a variety of scenarios (e.g., the violation of assumptions necessary for the linkage disequilibrium method such as random mating, no migration) (see Gilbert & Whitlock, 2015; Wang, 2016; Waples, 2016), the most commonly used estimator in our literature survey was the program LDNe (Waples & Do, 2008). This program makes use of linkage disequilibrium information to estimate N_e_/N_b_ and is one of the most accurate programs currently available (Gilbert & Whitlock, 2015). Furthermore, Bernos & Fraser, 2016 included a comparison between Colony (which uses the sibship method) and LDNe and found a stronger link between N_b_ and N when the LDNe method was used. To reduce potential bias associated with different estimators and/or methods, only N_b_ estimates obtained from LDNe were therefore used.

One potential issue that emerged with using data obtained from LDNe was the inconsistent use across studies of critical *p*‐value thresholds, which are used to exclude alleles with low frequencies from N_b_ estimation; low‐frequency alleles can cause bias in N_b_ estimates (Waples & Do, 2010). This problem is most apparent at low sample sizes, which require higher critical *p*‐values (Waples & Do, 2010); only N_b_ estimates derived from ≥30 samples were therefore retained (as in Johnstone, O'Connell, Palstra, & Ruzzante, 2012). Similarly, N_b_ estimates which included “infinity” as an upper confidence limit were excluded from the primary data set. When N_b_ and N_c_ estimates were contained in figures, the ImageJ program (Abràmoff, Magalhães, & Ram, 2005) was used to extract the data.

### 2.2 Quantitative analysis

The efficacy of predicting both N_c_ from N_b_ and N_b_ from N_c_ was assessed using generalized linear mixed effect models (GLMMs). To generate models from which we could derive predictions, we evaluated the effect of N_b_ on N_c_ (and vice versa) across multiple species using the MCMCglmm package (Hadfield, 2010) in R (version 2.13.0; R Development Core Team, 2016) with a Poisson distribution and a log‐link function given that census data represent counts of discrete individuals (as in Bernos & Fraser, 2016). MCMC chains were run for 1,000,000 iterations with a “burn in” period of 100,000 and thinning intervals of 50; the posterior distribution was therefore sampled > 10,000 times to obtain model parameters and associated 95% posterior density credible intervals (CI). Posterior traces and autocorrelation values were examined visually to evaluate and verify model convergence and mixing. The default (weakly informative) priors were used for all models.

Posterior modes for N_c_ or N_b_ were calculated from models in which N_b_ or N_c_ (respectively) was included as a continuous fixed effect and “species” was included as a categorical fixed effect. An interaction between both fixed effects terms was also included. Population, study and year‐within‐study terms were included as random effects to account for issues of nonindependence in the data. The year random effect was nested within study except when studies were conducted by the same group of researchers on the same populations, in which case year was nested across the relevant studies. Heterogeneous variances for the residuals were specified using the *idh* function; residual error variance was allowed to differ for each level of the species variable.

As population size becomes large, it becomes increasingly difficult to confidently estimate Nb or Ne (Waples & Do, 2010). Models were also fitted that allowed us to explore whether residual variance changed with the fixed effect population size (N_b_ or N_c_) variable. This was accomplished by fitting an observation‐level random effect of the form “idh(species:sqrt(1/ln(N_x_)):units” (when testing if variance decreased with the relevant population size variable, i.e., N_c_ or N_b_) or “idh(species:sqrt(N_x_)):units” (when testing if variance increased with the relevant population size variable) (as in Wood, Yates, & Fraser, 2016). Significance of this term was evaluated by comparing 95% CIs of N_x_‐related residual error estimates at five population sizes representative of the gradients present in our data set: 20, 50, 100, 300 and 600 for models predicting N_c_ from N_b_, and 50, 100, 500, 1 000 and 10,000 for models predicting N_b_ from N_c_. If CIs for the N_x_‐related residual variances overlapped between all representative population sizes, the heteroscedastic error term was subsequently removed.

Model performance was evaluated by calculating both marginal and conditional R^2^ (Nakagawa & Schielzeth, 2013); slight modifications to the code described in the paper had to be made to accommodate the modelling of heterogeneous residual variances at the species level. Multiple R^2^ values were computed for each model at the species level.

The efficacy and practicality of predicting N_c_ or N_b_ from a novel population (i.e., with random effects marginalized) was evaluated by examining 95% prediction intervals generated for each model across a gradient of N_c_ (when predicting N_b_) or N_b_ (when predicting N_c_). For most natural populations, N_b_ (or N_e_) is almost always less than N_c_ (Kalinowski & Waples, 2002; Waples et al., 2013). Hence, when predicting N_b_ from N_c_ for natural populations, biologically meaningful and informative predicted values should typically fall within the predictor N_c_ value and 0. If upper N_b_ prediction interval values were greater than the predictor N_c_ values used to obtain them, the upper prediction intervals were considered fundamentally uninformative. When predicting N_b_ from N_c_, lower 95% prediction interval values were considered “informative” only when they did not include (or were extremely close to) zero. When predicting N_c_ from N_b_, meaningful predicted values could include any value greater than the predictor N_b_ value; both upper and lower prediction interval values were considered “informative” at a given size only when they were greater than the predictor N_b_ value used to obtain them.

A supplementary analysis was conducted that predicted N_c_ from the lower N_b_ CI reported in each paper as these are relevant for many conservation situations. Namely, when populations are difficult to sample effectively (i.e., populations are too large or individual samples are difficult to obtain), it can be challenging to obtain bounded N_b_ point estimates, in which case lower CI may be more informative (Waples & Do, 2010). Using exclusively lower CI estimates allowed us to incorporate N_b_ estimates that had infinite upper CI, which increased the number of estimates in the data set by 42. However, prediction intervals calculated from N_b_ lower CI were always wider than models generated from point estimates (Figure S1); these models were therefore not reported.

## 3 RESULTS

### 3.1 Literature review

Of the 209 papers reviewed on N_b_/N_c_ across taxa, 11 contained data that met inclusion criteria. The final data set contained 144 individual N_b_/N_c_ estimates from 40 populations of three species: brook trout (15 populations), Atlantic salmon (14 populations) and Chinook salmon (11 populations) (Table 1). Any duplicate N_b_/N_c_ estimates across studies on the same populations were removed from the data set. No other species had three or more populations for which N_b_ and N_c_ data had been estimated in adequate quantities (i.e., ≥10 data points total). N_b_/N_c_ estimates for the three salmonid species included in this analysis were typically obtained across multiple years of sampling involving the genotyping of thousands of individuals; they represent the best data presently available in the literature for examining the predictive capacity of N_b_ to predict N_c_ (or vice versa).

**Table 1:** Published studies examining N_b_/N_c_ relationships among the three study species

Authors (Year)	Species	Number of Populations	Total N_b_/N_c_ estimates
Johnstone et al. (2012)	*Salmo salar*	1	8
Palstra, O'Connell, and Ruzzante (2009)	*Salmo salar*	2	2
Perrier, Normandeau, Dionne, Richard, & Bernatchez, (2014)	*Salmo salar*	1	1
Perrier et al. (2016)	*Salmo salar*	9	23
Bernos et al. (In revision)^a^	*Salmo salar*	2	4
Ferchaud et al. (2016)	*Salmo salar*	9	19
Whiteley et al. (2015)	*Salvelinus fontinalis*	2	12
Bernos and Fraser (2016)	*Salvelinus fontinalis*	11	31
Ruzzante et al. (2016)	*Salvelinus fontinalis*	2	2
Van Doornik et al. (2011)	*Oncorhynchus tshawytscha*	5	15
Van Doornik et al. (2013)	*Oncorhynchus tshawytscha*	6	27
	Overall totals	40^b^	144

a See Appendix S1.

b Some populations were examined more than once across studies.

### 3.2 Predicting N_b_ from N_c_

No evidence was found that residual error exhibited heteroscedasticity associated with N_c_. Estimates of residual variance did not change with N_c_; 95% CIs for residual error estimates overlapped for all population size ranges compared (Appendix S2). The heteroscedastic error term was therefore dropped from all subsequent analyses.

**Figure 1:**
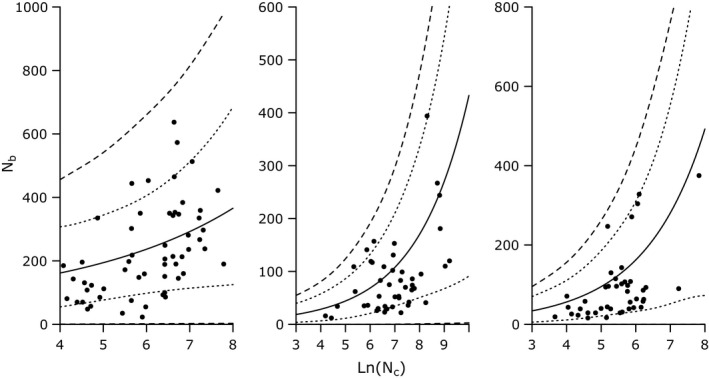
Relationship predicting N_b_ from N_c_ in Atlantic salmon (a), brook trout (b) and Chinook salmon (c). Dotted lines represent 95% credible intervals; dashed lines represent 95% prediction intervals

The slope of the relationship predicting N_b_ from N_c_ differed significantly between some species. Slope estimates were significantly or marginally lower for Atlantic salmon relative to Chinook salmon (*P*_mcmc_ = 0.0413, Table 2) and brook trout (*P*_mcmc_ = 0.076, Table 2), respectively; 95% CIs for estimated differences barely overlapped zero. The slope of the relationship predicting N_b_ from N_c_ differed marginally from zero for Atlantic salmon (*P*_mcmc_ = 0.078, Table 3, Figure 1), whereas posterior mode slope estimates for brook trout and Chinook salmon were significant and positive with CIs not overlapping zero (*P*_mcmc_ < 0.001 and *P*_mcmc _< 0.001, Table 3, Figure 1). Slope estimates did not differ between brook trout and Chinook salmon (*P*_mcmc_ = 0.563, Table 2).

**Table 2:** Between‐species slope estimate contrasts and 95% credible intervals when predicting N_b_ from N_c_ and N_c_ from N_b_

N_b_ from N_c_	Estimate	N_c_ from N_b_	Estimate
Contrast		Contrast	
AS vs CS	−0.336 (−0.658, −0.016)	AS vs CS	−0.518 (−0.895, −0.102)
AS vs BT	−0.269 (−0.510, 0.031)	AS vs BT	−0.488 (−0.945, −0.037)
BT vs CS	−0.066 (−0.377, 0.189)	BT vs CS	−0.127 (−0.557, 0.519)

AS, Atlantic salmon; CS, chinook salmon; BT, brook trout.

**Table 3:** Slope and intercept estimates with 95% credible intervals for models predicting N_b_ from N_c_ and N_c_ from N_b_ for three salmonid species

Species	Intercept	Slope	Marginal R^2^	Conditional R^2^
N_b_ from N_c_				
Atlantic salmon	3.705 (2.335, 5.266)	0.195 (−0.029, 0.429)	0.424	0.857
Brook trout	0.932 (−0.329, 2.480)	0.449 (0.278, 0.611)	0.394	0.865
Chinook salmon	1.200 (−0.411, 2.761)	0.528 (0.303, 0.765)	0.343	0.737
N_c_ from N_b_				
Atlantic salmon	5.821 (4.655, 7.013)	0.067 (−0.141, 0.272)	0.376	0.941
Brook trout	4.932 (2.824, 6.449)	0.590 (0.133, 0.976)	0.376	0.902
Chinook salmon	2.992 (1.537, 4.527)	0.558 (0.236, 0.902)	0.321	0.856

The N_c_ and species terms accounted for 34%–42% of the variation present in the data, depending on species; the population, study and year random effects terms accounted for 39%–47% of the variation (Table 3).

Prediction intervals for Atlantic salmon were uninformative as a result of a lack of a significant relationship predicting N_b_ from N_c_ (i.e., slope estimate CIs overlapped zero). Lower 95% prediction intervals for brook trout and Chinook salmon were uninformative; they included (or were extremely close to) zero for both species (Figure 1). Upper 95% prediction interval values were informative for most N_c_ values for brook trout: upper N_b_ prediction intervals were lower than predictor N_c_ values for census sizes greater than approximately 100 individuals. Chinook salmon upper prediction interval values were meaningful only for large N_c_ values; upper 95% prediction interval values were lower than predictor N_c_ values for census sizes greater than approximately 650 individuals.

### 3.3 Predicting N_c_ from N_b_

No evidence was found that residual error exhibited heteroscedasticity associated with N_b_. The CIs for residual error estimates also overlapped for all population size ranges compared (Appendix S2). The heteroscedastic error term was therefore dropped from all subsequent analyses.

**Figure 2:**
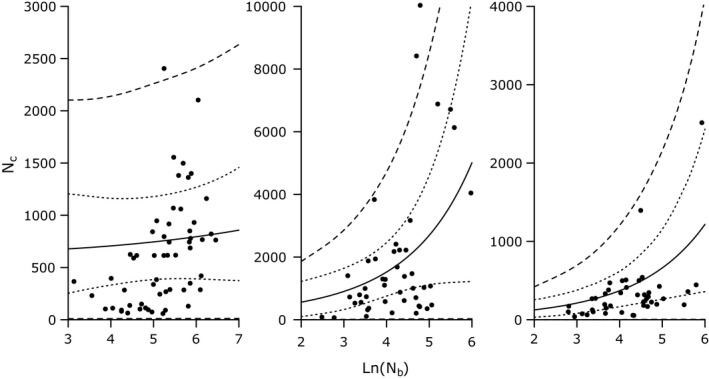
Relationship predicting N_c_ from N_b_ in Atlantic salmon (a), brook trout (b) and Chinook salmon (c). Dotted lines represent 95% credible intervals; dashed lines represent 95% prediction intervals

The slope of the relationship predicting N_c_ from N_b_ also differed significantly between some species. The slope estimates for Atlantic salmon were significantly lower than for Chinook salmon (*P*_mcmc_ = 0.011, Table 2) and brook trout (*P*_mcmc_ = 0.031, Table 2). The slope of the relationship predicting N_c_ from N_b_ did not differ from zero for Atlantic salmon (*P*_mcmc_ = 0.550, Table 3, Figure 2). Posterior mode slope estimates for both brook trout and Chinook salmon were again significantly positive, and CIs did not overlap zero (*P*_mcmc_ = 0.009 and *P*_mcmc_ = 0.001, Table 3, Figure 2); slope estimates also did not differ between brook trout and Chinook salmon (*P*_mcmc_ = 0.941, Table 2).

The N_b_ and species terms accounted for 32%–38% of the variation present in the data, depending on species; the population, study and year random effects terms accounted for 53%–57% of the variation.

Prediction intervals for Atlantic salmon were uninformative as a result of a lack of a significant relationship predicting N_c_ from N_b_ (i.e., slope estimate CIs overlapped zero). Lower 95% prediction intervals for brook trout and Chinook salmon were uninformative; they included (or were extremely close to) zero for both species (Figure 2). Upper 95% prediction interval values were meaningful for all N_c_ values for both species: upper prediction intervals for N_c_ were always greater than predictor N_b_ values for all population sizes.

## 4 DISCUSSION

### 4.1 Using N_c_ to predict N_b_ or using N_b_ to predict N_c_

Recent studies have suggested that N_b_ and N_c_ were correlated among intraspecific populations and that one could be used to predict the other if N_b_/N_c_ relationships were well characterized for a particular species (Bernos & Fraser, 2016; Ferchaud et al., 2016; Osborne et al., 2010). To formally test this hypothesis, we modelled the relationship between N_b_ and N_c_ using a database of 40 populations from three salmonid fishes and generated prediction intervals using those models to determine efficacy of predicting one population size variable from the other. The 95% prediction intervals for some species provided potential maximum thresholds for some population size variables. For example, a brook trout population with an N_c_ of approximately 1,000 is not likely to have an N_b_ higher than 300. However, the practical usefulness of this upper threshold varies depending on the species and the estimated variable.

Brook trout and Chinook salmon had potentially informative and biologically meaningful upper prediction intervals for N_c_ when predicted from N_b_. Upper prediction thresholds, however, were up to almost two orders of magnitude larger than the predictor N_b_ values, placing them on the extreme end of N_b_/N_c_ ratios documented in wild salmonid populations (Palstra & Fraser, 2012). Furthermore, while “informative” upper thresholds for N_b_ can be predicted from moderate and large N_c_ values for brook trout and Chinook salmon, these thresholds may not be informative from a practical management standpoint because, from a conservation genetics standpoint, N_e_ is often the variable of more interest. Both N_e_ and N_b_ are likely to be less than N_c_ in natural populations (Waples et al., 2013); the criteria for biologically meaningful predicted N_b_ values would, however, be even more stringent when translating predicted N_b_ values to N_e_ values given that N_b_ is typically less than N_e_. N_b_ in brook trout, for example, can range from 0.34 to 0.68 of N_e_, depending on the conversion methodology used (Bernos & Fraser, 2016).

It is also unsurprising that N_b_ upper prediction interval values overlapped with predictor N_c_ values at small N_c_ in brook trout and small‐to‐moderate N_c_ in Chinook salmon; as N_c_ increases, the N_b_/N_c_ ratio tends to decrease because of density‐dependent effects on reproduction (Bernos & Fraser, 2016; Ferchaud et al., 2016; Whiteley et al., 2015). The models also did not accurately provide minimum prediction thresholds for both population size variables; for all species, lower prediction intervals at all N_b_ or N_c_ sizes either included or were extremely close to zero across all population sizes examined.

Recent empirical studies have shown that changes in N_b_ do not always track changes in N_c_ within a population over time (Bernos & Fraser, 2016). Primary studies also reported that spatial variation among populations in N_b_/N_c_ ratios was approximately twofold greater than temporal variation within populations for two of the three species in our synthesis (Bernos & Fraser, 2016; Ferchaud et al., 2016). Similarly, study, population and year level random effects accounted for substantial amounts of variation in our analysis (between 39%–57%). This variability in N_b_/N_c_ is likely a result of several biological processes acting differentially and simultaneously within and among populations, including the degree of population connectivity (Fraser, Lippé, & Bernatchez, 2004; Gomez‐Uchida et al., 2013; Lamy, Pointier, Jarne, & David, 2012), environmental conditions (Bernos & Fraser, 2016; Whiteley et al., 2015) or ecological differences (Belmar‐Lucero et al., 2012; Waples et al., 2013). Such population variability present in both N_b_ and N_c_ measurements probably affected the accuracy and precision of predictions, limiting the utility of the models for predicting N_b_ or N_c_ for novel, nonsampled populations.

### 4.2 Relationship between N_b_ and N_c_ among three salmonid species

Another primary study objective was to assess whether the trajectory and magnitude of the relationship between N_b_ and N_c_ differed among taxonomically related species; our results provide mixed support for this at the Salmoninae subfamily level. A general positive correlation between N_b_ and N_c_ was observed in brook trout and Chinook salmon: larger populations tend to have larger N_b_. However, the slope estimates for Atlantic salmon predicting N_c_ from N_b_ or N_b_ from N_c_ were either not significantly different from zero or only marginally different from zero. Therefore, (i) taxonomically related species should not be assumed to exhibit similar N_b_/N_c_ ratios; and (ii) ecological and life‐history characteristics of naturally spawning Atlantic salmon could buffer small populations against a loss of genetic diversity.

Reproductive life histories differ markedly among salmonids. While male brook trout exhibit variable ages at maturity (Hutchings, 1993) and male Chinook salmon exhibit alternative reproductive phenotypes (Berejikian et al., 2010), male Atlantic salmon exhibit one of two extreme reproductive phenotypes: an early maturing freshwater phenotype (1–2 years of age) or an anadromous phenotype (typically 4–6 years of age) (Hutchings & Jones, 1998; Myers, Hutchings, & Gibson, 1986). In some populations, up to 80% of males delay or forgo oceanic migration to mature in freshwater (Myers et al., 1986) at a size much smaller than their anadromous conspecifics (Hutchings & Myers, 1988). The presence of the early maturation phenotype is well known to have a positive influence on N_e_ by balancing sex ratios, decreasing variance in reproductive success and increasing outbreeding between cohorts within a population (Johnstone et al., 2012; Jones & Hutchings, 2001; Perrier et al., 2014; Saura, Caballero, Caballero, & Morán, 2008).

The primary literature N_c_ estimates excluded early maturation phenotypes (“parr”) in all but two (landlocked) Atlantic salmon populations. Most N_c_ estimates are actually estimates of anadromous adults only and therefore underestimate the number of reproductive individuals within a population (Myers, 1984; Perrier et al., 2014). This very likely explains the lack of relationship between N_c_ and N_b_ for Atlantic salmon; observed N_b_/N_c_ ratios are probably upwardly biased because the male alternative phenotype may buffer N_b_ estimates when male anadromous numbers are small (Ferchaud et al., 2016; Johnstone et al., 2012). Future research on any species should include all reproductive phenotypes when estimating N_c_.

N_b_/N_c_ relationships were similar in brook trout and Chinook salmon, with N_b_ tending to increase at a similar rate with N_c_. These species have substantial differences in life histories (e.g., semelparity vs. iteroparity, obligate vs. facultative anadromy), but their spawning behaviour can be similar. Both, for example, prefer spawning habitat with hypoheic upwelling (Curry & Noakes, 1995; Geist, 2000; Geist & Dauble, 1998) and spawn at high densities; brook trout have been observed to exhibit aggregate spawning (Belmar‐Lucero et al., 2012; Blanchfield & Ridgway, 1997) and Chinook salmon spawn in clusters at densities much higher than Atlantic salmon (Fleming, 1998; Geist & Dauble, 1998). As N_c_ increases within populations in both species similar density‐dependent issues may emerge (i.e., mate competition, nest superimposition) and affect N_b_.

Overall, the among species comparisons suggest that extrapolating N_b_ or N_c_ estimates from N_b_/N_c_ curves for species related at the family/subfamily taxonomic level may, in some cases, over‐ or underestimate population size estimates; mixed evidence was found that N_b_/N_c_ relationships differed between these species, with observed differences likely a result of different species‐level life‐history characteristics. If N_b_/N_c_ relationships for a taxonomically related species are used as “proxy” population parameters for another “data‐deficient” species, careful consideration should be taken to evaluate life‐history and behavioural similarities to determine whether such an extrapolation is valid or meaningful.

### 4.3 Cost‐benefit consideration in quantifying N_b_ or N_c_ to infer the other

Conservation resources are often limited; it is therefore important to consider the relative costs of quantifying N_c_ and N_b_ in wild populations given the degree of uncertainty in converting one to the other. To help other researchers considering similar research projects, we provided an example of the comparative costs of estimating N_c_ and N_b_ in a series of stream brook trout populations of varying size from Cape Race, Newfoundland, Canada (Table 4). This was based on one of the largest empirical studies of N_b_/N_c_ to date (Bernos & Fraser, 2016; see Table 1). Intriguingly, the relative costs of quantifying N_c_ and N_b_ were very similar. Costs unsurprisingly increased with increasing population size: in general, more labour resources were required to estimate N_c_ and more consumables were required to estimate N_b_ using molecular markers. Given this, the choice of estimating N_c_ or N_b_ may depend largely on how much confidence one desires in estimating either variable specifically while balancing other considerations. For example, at Cape Race estimating N_c_ with accuracy and precision is feasible but can be invasive, requiring the tagging of many adults within streams (especially for large populations). Conversely, estimating N_b_ is arguably less invasive in relying on sampling juvenile cohorts that naturally experience density dependence, but these N_b_ estimates may only translate into maximum estimates of N_c_.

**Table 4:** Example cost‐benefit trade‐offs associated with estimating N_c_ and N_b_ in wild populations, based on one of the largest N_b_/N_c_ studies to date conducted on brook trout occupying small streams in Cape Race, Newfoundland, Canada (Bernos & Fraser, 2016). Expenses are approximate and in CDN dollars

Expense	Small population	Medium population	Large population
	N_c_ = 50–500	N_c_ = 500–1,500	N_c_ = 1500–10000
N_c_ estimation from mark–recapture			
Field labour (person days)	$180 (1.2)	$360 (2.4)	$600 (4.0)
Equipment use and maintenance demands	$35	$50	$95
Office labour (person days)	$20 (0.13)	$20 (0.13)	$20 (0.13)
Miscellaneous field expenses^a^	$200	$225	$715
Total cost, N_c_ estimation	$435	$655	$1,460
N_b_ estimation using molecular markers			
Field labour (person days)	$75 (0.5)	$150 (1.0)	$225 (1.5)
Equipment use and maintenance demands	$65	$110	$150
Molecular laboratory and office labour (person days)	$180 (1.20)	$255 (1.70)	$330 (2.20)
Molecular consumables^b^	$240	$440	$640
Total cost, N_b_ estimation	$560	$955	$1345

a Does not include travel expenses to/from field site (gas/food/accommodation), nor travel expenses for the marking event (these would be equivalent for N_b_ and N_c_ estimation).

b Based on 10–15 microsatellite loci, and sample sizes of *n* = 35, 65, and 95 for small, medium and large populations, respectively.

### 4.4 Future research

The number of populations with data available for each species in our models was modest (11–15 per species, limited to the *Salmoninae* subfamily). The species examined in this study (salmonids) may share life‐history traits that could potentially obscure the relationship between Nb and Nc. Salmonids, for example, are characterized by type‐III survival curves; species with high fecundity and juvenile mortality typically exhibit low N_e_/N_c_ ratios (Palstra & Ruzzante, 2008). The salmonid species examined also exhibit high variance in reproductive success (Blanchfield, Ridgway, & Wilson, 2003; Tentelier et al., 2016). Relationships between N_b_ and N_c_ for species with higher N_b_/N_c_ ratios or lower variance in N_b_ over time could be stronger. This review examined data for all taxa, but sufficient data were available only for species from the Salmoninae subfamily; unfortunately, the data necessary to examine N_b_/N_c_ relationships among other taxonomic groups with differing life‐history characteristics are not available in the scientific literature at this time.

While several other species (both salmonid and nonsalmonid) did have studies in which both N_b_ and N_c_ were estimated (see Appendix S3) they were excluded from our final data set for three reasons: (i) population size variables were only estimated for one or two populations across studies in each species; (ii) adequate data did not exist to generate robust species curves (i.e., ≥10 datapoints); or (iii) LDNe was not used to obtain N_b_ estimates. As further studies examine N_b_/N_c_ relationships within a variety of taxa, it may be possible to generate more robust predictive models and increase their practical utility for conservation purposes.

This study focused partially on the practicality of predicting N_b_ or N_c_ for novel, previously unsampled populations based on relationships generated from recently published data. While these predictive models were somewhat limited in their practical applications, it may still be possible to use these models to reliably infer one population size variable from the other for populations with well‐established baseline data. Population and temporal model terms often account for a significant component of the variation observed in population size terms (Ferchaud et al., 2016); with enough temporal data, it may be possible to reliably track changes in one population size variable through the other (but see Bernos & Fraser, 2016). Although outside the scope of this study, future research could examine under what conditions this could be reliably carried out. For example, how many years of historical data are necessary to reliably track a given population? Are N_b_/N_c_ relationships in some species more variable over time than others? Are certain ecological or life‐history traits among populations associated with more stable N_b_/N_c_ relationships?

The extent to which the N_b_/N_c_ relationships explored herein apply to differing ecotypes of the explored species is also unknown. Salmonids are an extremely plastic taxa; many species have multiple life‐histories and/or inhabit a wide range of habitats. The brook trout populations represented in this study, for example, are largely lentic; whether the modelled N_b_/N_c_ relationships could be extrapolated to lotic or anadromous populations remains undetermined.

Finally, we found no evidence for heteroscedasticity in any of the modelled N_b_/N_c_ relationships, although there have been indications of this across population size gradients in studies with a large number of populations (Bernos & Fraser, 2016). Therefore, future studies are encouraged to continue to account for this potential heteroscedasticity, particularly given that it becomes increasingly difficult to estimate genetic population size variables (N_b_, N_e_) for large populations (Waples & Do, 2010).

## 5 CONCLUSIONS

Although estimating the maximum number of adults present in a given population could help guide management and conservation decisions, the upper prediction intervals determined herein generally represented documented taxonomic extremes for N_b_/N_c_ ratios in salmonids and lower prediction intervals were largely uninformative; predicting a precise N_b_ or N_c_ estimate for a novel population based off of a single population size variable is, with current data available, not realistically possible. While N_c_ prediction intervals generated from N_b_ estimates were marginally worse than prediction intervals in salmonids generated from other molecular data (e.g., eDNA in Baldigo et al., 2017), realizing the full potential of the anticipated conservation applications of genetic techniques to predict and estimate N_c_ (e.g., Luikart et al., 2010) will realistically require the accumulation of more data.

Molecular technologies and methods are rapidly advancing and could represent a practical means of estimating N_c_ in the future. However, researchers should be cognizant of the limitations of using one population size variable to infer the other; researchers and/or managers should, whenever possible, focus efforts on quantifying the population size variable of interest except when the costs/logistics of measuring that variable are prohibitive. Further research is also necessary to determine whether less variable relationships exist between N_b_ and N_c_ for other taxonomic groups with differing life‐history characteristics than salmonids.

## 6 DATA ARCHIVING STATEMENT

Data for this study are available from the Dryad Digital Repository: https://doi.org/10.5061/dryad.136bm.

## Supporting information


** **

** **

** **

** **

## References

[eva12496-bib-0001] Abràmoff, M. D. , Magalhães, P. J. , & Ram, S. J. (2005). Image processing with ImageJ Part II. Biophotonics International, 11, 36–43. https://doi.org/10.1117/1.3589100.

[eva12496-bib-0002] Baldigo, B. P. , Sporn, L. A. , George, S. D. , & Ball, J. A. (2017). Efficacy of Environmental DNA to Detect and Quantify Brook Trout Populations in Headwater Streams of the Adirondack Mountains New York. Transactions of the American Fisheries Society, 146, 99–111. https://doi.org/10.1080/00028487.2016.1243578.

[eva12496-bib-0003] Beebee, T. J. C. (2009). A comparison of single‐sample effective size estimators using empirical toad (Bufo calamita) population data: Genetic compensation and population size‐genetic diversity correlations. Molecular Ecology, 18, 4790–4797. https://doi.org/10.1111/j.1365-294X.2009.04398.x. 19863715 10.1111/j.1365-294X.2009.04398.x

[eva12496-bib-0004] Belmar‐Lucero, S. , Wood, J. L. A. , Scott, S. , Harbicht, A. B. , Hutchings, J. A. , & Fraser, D. J. (2012). Concurrent habitat and life history influences on effective/census population size ratios in stream‐dwelling trout. Ecology and Evolution, 2, 562–573. https://doi.org/10.1002/ece3.196. 22822435 10.1002/ece3.196 PMC3399145

[eva12496-bib-0005] Berejikian, B. A. , Van Doornik, D. M. , Endicott, R. C. , Hoffnagle, T. L. , Tezak, E. P. , Moore, M. E. , & Atkins, J. (2010). Mating success of alternative male phenotypes and evidence for frequency‐dependent selection in Chinook salmon, Oncorhynchus tshawytscha. Canadian Journal of Fisheries and Aquatic Science, 67, 1933–1941. https://doi.org/10.1139/F10-112.

[eva12496-bib-0006] Bernos, T. A. , & Fraser, D. J. (2016). Spatiotemporal relationship between adult census size and genetic population size across a wide population size gradient. Molecular Ecology, 25, 4472–4487. https://doi.org/10.1111/mec.13790. 27483203 10.1111/mec.13790

[eva12496-bib-0501] Bernos, T. B. , Yates, M. C. , & Fraser, D. J. (In revision). Fine‐scale differences in genetic and census population size ratios between two stream fishes.

[eva12496-bib-0007] Blanchfield, P. J. , & Ridgway, M. S. (1997). Reproductive timing and use of redd sites by lake‐spawning brook trout (Salvelinus fontinalis). Canadian Journal of Fisheries and Aquatic Science, 54, 747–756. https://doi.org/10.1139/f96-344.

[eva12496-bib-0008] Blanchfield, P. J. , Ridgway, M. S. , & Wilson, C. C. (2003). Breeding success of male brook trout (Salvelinus fontinalis) in the wild. Molecular Ecology, 12, 2417–2428. https://doi.org/10.1046/j.1365-294X.2003.01917.x. 12919479 10.1046/j.1365-294x.2003.01917.x

[eva12496-bib-0009] Curry, R. A. , & Noakes, D. L. G. (1995). Groundwater and the selection of spawning sites by brook trout (Salvelinus fontinalis). Canadian Journal of Fisheries and Aquatic Science, 52, 1733–1740. https://doi.org/10.1139/f95-765.

[eva12496-bib-0011] Ferchaud, A.‐L. , Perrier, C. , April, J. , Hernandez, C. , Dionne, M. , & Bernatchez, L. (2016). Making sense of the relationships between Ne, Nb and Nc towards defining conservation thresholds in Atlantic salmon (Salmo salar). Heredity, 117, 1–11. https://doi.org/10.1038/hdy.2016.62. 27530910 10.1038/hdy.2016.62 PMC5026759

[eva12496-bib-0012] Fleming, I. A. (1998). Pattern and variability in the breeding system of Atlantic salmon (Salmo salar), with comparisons to other salmonids. Canadian Journal of Fisheries and Aquatic Science, 55, 59–76. https://doi.org/10.1139/cjfas-55-S1-59.

[eva12496-bib-0013] Fraser, D. J. , Calvert, A. M. , Bernatchez, L. , & Coon, A. (2013). Multidisciplinary population monitoring when demographic data are sparse: A case study of remote trout populations. Ecology and Evolution, 3, 4954–4969. https://doi.org/10.1002/ece3.871. 24455128 10.1002/ece3.871 PMC3892360

[eva12496-bib-0014] Fraser, D. J. , Lippé, C. , & Bernatchez, L. (2004). Consequences of unequal population size, asymmetric gene flow and sex‐biased dispersal on population structure in brook charr (Salvelinus fontinalis). Molecular Ecology, 13, 67–80. https://doi.org/10.1046/j.1365-294X.2003.02038.x. 14653789 10.1046/j.1365-294x.2003.02038.x

[eva12496-bib-0016] Geist, D. R. (2000). Hyporheic discharge of river water into fall chinook salmon (*Oncorhynchus tshawytscha*) spawning areas in the Hanford Reach, Columbia River. Canadian Journal of Fisheries and Aquatic Science, 57, 1647–1656. https://doi.org/10.1139/cjfas-57-8-1647.

[eva12496-bib-0017] Geist, D. R. , & Dauble, D. D. (1998). Redd site selection and spawning habitat use by fall chinook salmon: The importance of geomorphic features in large rivers. Environmental Management, 22, 655–669. https://doi.org/10.1007/s002679900137. 9680535 10.1007/s002679900137

[eva12496-bib-0018] Gilbert, K. J. , & Whitlock, M. C. (2015). Evaluating methods for estimating local effective population size with and without migration. Evolution; International Journal of Organic Evolution, 69, 2154–2166. https://doi.org/10.1111/evo.12713. 26118738 10.1111/evo.12713

[eva12496-bib-0019] Gomez‐Uchida, D. , Palstra, F. P. , Knight, T. W. , & Ruzzante, D. E. (2013). Contemporary effective population and metapopulation size (Ne and meta‐Ne): Comparison among three salmonids inhabiting a fragmented system and differing in gene flow and its asymmetries. Ecology and Evolution, 3, 569–580. https://doi.org/10.1002/ece3.485. 23532448 10.1002/ece3.485 PMC3605847

[eva12496-bib-0020] Guschanski, K. , Vigilant, L. , McNeilage, A. , Gray, M. , Kagoda, E. , & Robbins, M. M. (2009). Counting elusive animals: Comparing field and genetic census of the entire mountain gorilla population of Bwindi Impenetrable National Park, Uganda. Biological Conservation, 142, 290–300. https://doi.org/10.1016/j.biocon.2008.10.024.

[eva12496-bib-0021] Hadfield, J. D. (2010). MCMC methods for multi‐response generalized linear mixed models: The MCMCglmm R package. Journal of Statistical Software, 33, 1–22. https://doi.org/10.1002/ana.22635. 20808728 PMC2929880

[eva12496-bib-0022] Hoehn, M. , Gruber, B. , Sarre, S. D. , Lange, R. , & Henle, K. (2012). Can genetic estimators provide robust estimates of the effective number of breeders in small populations? PLoS ONE, 7, e48464 https://doi.org/10.1371/journal.pone.0048464. 23139784 10.1371/journal.pone.0048464 PMC3491051

[eva12496-bib-0023] Hutchings, J. A. (1993). Adaptive life histories effected by age‐specific surivival and growth rate. Ecology, 74, 673–684. https://doi.org/10.2307/1940795.

[eva12496-bib-0024] Hutchings, J. A. , & Jones, M. E. B. (1998). Life history variation and growth rate thresholds for maturity in Atlantic salmon, Salmo salar. Canadian Journal of Fisheries and Aquatic Science, 55, 22–47. https://doi.org/10.1139/cjfas-55-S1-22.

[eva12496-bib-0025] Hutchings, J. A. , & Myers, R. A. (1988). Mating success of alternative maturation phenotypes in male Atlantic salmon, Salmo salar. Oecologia, 75, 169–174. https://doi.org/10.1007/BF00378593. 28310830 10.1007/BF00378593

[eva12496-bib-0026] Johnstone, D. L. , O'Connell, M. F. , Palstra, F. P. , & Ruzzante, D. E. (2012). Mature male parr contribution to the effective size of an anadromous Atlantic salmon (Salmo salar) population over 30 years. Molecular Ecology, 22, 2394–2407. https://doi.org/10.1111/mec.12186. 10.1111/mec.12186 23317429

[eva12496-bib-0027] Jones, M. W. , & Hutchings, J. A. (2001). The influence of male pair body size and mate competition on fertilization success and effective population size in Atlantic salmon. Heredity, 86, 675–684. https://doi.org/10.1046/j.1365-2540.2001.t01-1-00880.x. 11595048 10.1046/j.1365-2540.2001.00880.x

[eva12496-bib-0028] Kalinowski, S. T. , & Waples, R. S. (2002). Relationship of Effective to Census Size in Fluctuating Populations. Conservation Biology, 16, 129–136. https://doi.org/10.1046/j.1523-1739.2002.00134.x. 10.1046/j.1523-1739.2002.00134.x 35701960

[eva12496-bib-0029] Lacoursière‐Roussel, A. , Côté, G. , Leclerc, V. , Bernatchez, L. , & Cadotte, M. (2016). Quantifying relative fish abundance with eDNA: A promising tool for fisheries management. Journal of Applied Ecology, 53, 1148–1157. https://doi.org/10.1111/1365-2664.12598.

[eva12496-bib-0030] Lamy, T. , Pointier, J. P. , Jarne, P. , & David, P. (2012). Testing metapopulation dynamics using genetic, demographic and ecological data. Molecular Ecology, 21, 1394–1410. https://doi.org/10.1111/j.1365-294X.2012.05478.x. 22332609 10.1111/j.1365-294X.2012.05478.x

[eva12496-bib-0031] Luikart, G. , Ryman, N. , Tallmon, D. A. , Schwartz, M. K. , & Allendorf, F. W. (2010). Estimation of census and effective population sizes: The increasing usefulness of DNA‐based approaches. Conservation Genetics, 11, 355–373. https://doi.org/10.1007/s10592-010-0050-7.

[eva12496-bib-0032] Myers, R. A. (1984). Demographic Consequences of Precocious Maturation of Atlantic Salmon (Salmo salar). Canadian Journal of Fisheries and Aquatic Science, 41, 1349–1353. https://doi.org/10.1139/f84-165.

[eva12496-bib-0033] Myers, R. A. , Hutchings, J. A. , & Gibson, R. J. (1986). Variation in male parr maturation within and among populations of Atlantic salmon, Salmo salar. Canadian Journal of Fisheries and Aquatic Science, 43, 1242–1248.

[eva12496-bib-0034] Nakagawa, S. , & Schielzeth, H. (2013). A general and simple method for obtaining R2 from generalized linear mixed‐effects models. Methods in Ecology and Evolution, 4, 133–142. https://doi.org/10.1111/j.2041-210x.2012.00261.x.

[eva12496-bib-0035] Osborne, M. J. , Davenport, S. R. , Hoagstrom, C. W. , & Turner, T. F. (2010). Genetic effective size, Ne, tracks density in a small freshwater cyprinid, Pecos bluntnose shiner (Notropis simus pecosensis). Molecular Ecology, 19, 2832–2844. https://doi.org/10.1111/j.1365-294X.2010.04695.x. 20579288 10.1111/j.1365-294X.2010.04695.x

[eva12496-bib-0036] Ovenden, J. R. , Blower, D. C. , Dudgeon, C. L. , Jones, A. T. , Buckworth, R. C. , Leigh, G. M. , … Bennett, M. B. (2016). Can genetic estimates of population size contribute to fisheries stock assessements? Journal of Fish Biology, 89(6), 2505–2518. https://doi.org/10.1111/jfb.13129. 27730623 10.1111/jfb.13129

[eva12496-bib-0037] Palstra, F. P. , & Fraser, D. J. (2012). Effective/census population size ratio estimation: A compendium and appraisal. Ecology and Evolution, 2, 2357–2365. https://doi.org/10.1002/ece3.329. 23139893 10.1002/ece3.329 PMC3488685

[eva12496-bib-0038] Palstra, F. P. , O'Connell, M. F. , & Ruzzante, D. E. (2009). Age structure, changing demography and effective population size in Atlantic salmon (Salmo salar). Genetics, 182, 1233–1249. https://doi.org/10.1534/genetics.109.101972. 19528328 10.1534/genetics.109.101972 PMC2728862

[eva12496-bib-0039] Palstra, F. P. , & Ruzzante, D. E. (2008). Genetic estimates of contemporary effective population size: What can they tell us about the importance of genetic stochasticity for wild population persistence? Molecular Ecology, 17, 3428–3447. https://doi.org/10.1111/j.1365-294X.2008.03842.x. 19160474 10.1111/j.1365-294x.2008.03842.x

[eva12496-bib-0040] Perrier, C. , April, J. , Cote, G. , Bernatchez, L. , & Dionne, M. (2016). Effective number of breeders in relation to census size as management tools for Atlantic salmon conservation in a context of stocked populations. Conservation Genetics, 17, 31–44. https://doi.org/10.1007/s10592-015-0758-5.

[eva12496-bib-0041] Perrier, C. , Normandeau, É. , Dionne, M. , Richard, A. , & Bernatchez, L. (2014). Alternative reproductive tactics increase effective population size and decrease inbreeding in wild Atlantic salmon. Evolutionary Applications, 7, 1094–1106. https://doi.org/10.1111/eva.12172. 25553070 10.1111/eva.12172 PMC4231598

[eva12496-bib-0042] R Development Core Team . (2016). R: A Language and Environment for Statistical Computing. Vienna, Austria: R Found Stat Comput 0:{ISBN} 3‐900051‐07‐0. https://doi.org/10.1038/sj.hdy.6800737

[eva12496-bib-0043] Ruzzante, D. E. , McCracken, G. R. , Parmelee, S. , Hill, K. , Corrigan, A. , MacMillan, J. , & Walde, S.J. (2016). Effective number of breeders, effective population size and their relationship with census size in an iteroparous species, Salvelinus fontinalis. Proceedings. Biological sciences, 283, 20152601 https://doi.org/10.1098/rspb.2015.2601. 26817773 10.1098/rspb.2015.2601 PMC4795031

[eva12496-bib-0044] Saura, M. , Caballero, A. , Caballero, P. , & Morán, P. (2008). Impact of precocious male parr on the effective size of a wild population of Atlantic salmon. Freshwater Biology, 53, 2375–2384. https://doi.org/10.1111/j.1365-2427.2008.02062.x.

[eva12496-bib-0045] Tallmon, D. A. , Gregovich, D. , Waples, R. S. , Scott Baker, C. , Jackson, J. , Taylor, B. L. , … Schwartz, M. K. (2010). When are genetic methods useful for estimating contemporary abundance and detecting population trends? Molecular Ecology Resources, 10, 684–692. https://doi.org/10.1111/j.1755-0998.2010.02831.x. 21565073 10.1111/j.1755-0998.2010.02831.x

[eva12496-bib-0046] Tentelier, C. , Lepais, O. , Larranaga, N. , Manicki, A. , Lange, F. , & Rives, J. (2016). Sexual selection leads to a tenfold difference in reproductive success of alternative reproductive tactics in male Atlantic salmon. Naturwissenschaften, 103, 47 https://doi.org/10.1007/s00114-016-1372-1. 27216174 10.1007/s00114-016-1372-1

[eva12496-bib-0047] Van Doornik, D. M. , Eddy, D. L. , Waples, R. S. , Boe, S. J. , Hoffnagle, T. L. , Berntson, E. A. , & Moran, P. (2013). Genetic Monitoring of Threatened Chinook Salmon Populations: Estimating Introgression of Nonnative Hatchery Stocks and Temporal Genetic Changes. North American Journal of Fisheries Management, 33, 693–706. https://doi.org/10.1080/02755947.2013.790861.

[eva12496-bib-0048] Van Doornik, D. M. , Waples, R. S. , Baird, M. C. , Moran, P. , & Berntson, E. A. (2011). Genetic Monitoring Reveals Genetic Stability within and among Threatened Chinook Salmon Populations in the Salmon River, Idaho. North American Journal of Fisheries Management, 31, 96–105. https://doi.org/10.1080/02755947.2011.562443.

[eva12496-bib-0049] Wang, J. (2005). Estimation of effective population sizes from data on genetic markers. Philosophical transactions of the Royal Society of London. Series B, Biological sciences, 360, 1395–1409. https://doi.org/10.1098/rstb.2005.1682. 16048783 10.1098/rstb.2005.1682 PMC1847562

[eva12496-bib-0050] Wang, J. (2016). A comparison of single‐sample estimators of effective population sizes from genetic marker data. Molecular Ecology, 25, 4692–4711. https://doi.org/10.1111/mec.13725. 27288989 10.1111/mec.13725

[eva12496-bib-0051] Waples, R. S. (2016). Making sense of genetic estimates of effective population size. Molecular Ecology, 25, 4689–4691. https://doi.org/10.1111/mec.13814. 27671356 10.1111/mec.13814

[eva12496-bib-0052] Waples, R. S. , & Antao, T. (2014). Intermittent breeding and constraints on litter size: Consequences for effective population size per generation (ne) and per reproductive cycle (nb). Evolution; International Journal of Organic Evolution, 68, 1722–1734. https://doi.org/10.1111/evo.12384. 24611912 10.1111/evo.12384

[eva12496-bib-0053] Waples, R. S. , & Do, C. (2008). LDNE: A program for estimating effective population size from data on linkage disequilibrium. Molecular Ecology Resources, 8, 753–756. https://doi.org/10.1111/j.1755-0998.2007.02061.x. 21585883 10.1111/j.1755-0998.2007.02061.x

[eva12496-bib-0054] Waples, R. S. , & Do, C. (2010). Linkage disequilibrium estimates of contemporary Ne using highly variable genetic markers: A largely untapped resource for applied conservation and evolution. Evolutionary Applications, 3, 244–262. https://doi.org/10.1111/j.1752-4571.2009.00104.x. 25567922 10.1111/j.1752-4571.2009.00104.x PMC3352464

[eva12496-bib-0055] Waples, R. S. , Luikart, G. , Faulkner, J. R. , & Tallmon, D. A. (2013). Simple life‐history traits explain key effective population size ratios across diverse taxa. Proceedings of the Royal Society B, 280, 20131339 https://doi.org/10.1098/rspb.2013.1339. 23926150 10.1098/rspb.2013.1339 PMC3757969

[eva12496-bib-0056] Whiteley, A. R. , Coombs, J. A. , Cembrola, M. , O'Donnell, M. J. , Hudy, M. , Nislow, K. H. , & Letcher, B. H. (2015). Effective number of breeders provides a link between interannual variation in stream flow and individual reproductive contribution in a stream salmonid. Molecular Ecology, 24, 3585–3602. https://doi.org/10.1111/mec.13273. 26080621 10.1111/mec.13273

[eva12496-bib-0057] Whiteley, A. R. , Coombs, J. A. , Hudy, M. , Robinson, Z. , Colton, A. R. , Nislow, K. H. , & Letcher, B. H. (2013). Fragmentation and patch size shape genetic structure of brook trout populations. Canadian Journal of Fisheries and Aquatic Science, 688, 678–688. https://doi.org/10.1139/cjfas-2012-0493.

[eva12496-bib-0058] Wood, J. L. A. , Yates, M. C. , & Fraser, D. J. (2016). Are heritability and selection related to population size in nature? Meta‐analysis and conservation implications. Evolutionary Applications, 9, 640–657. https://doi.org/10.1111/eva.12375. 27247616 10.1111/eva.12375 PMC4869407

