# Supplementary figures and images for: A critical assessment of estimating census population size from genetic population size (or vice versa) in three fishes
Source: Evol Appl. 2017 Jul 4;10(9):935–45. doi: 10.1111/eva.12496 (PMC5680432; doi:10.1111/eva.12496)

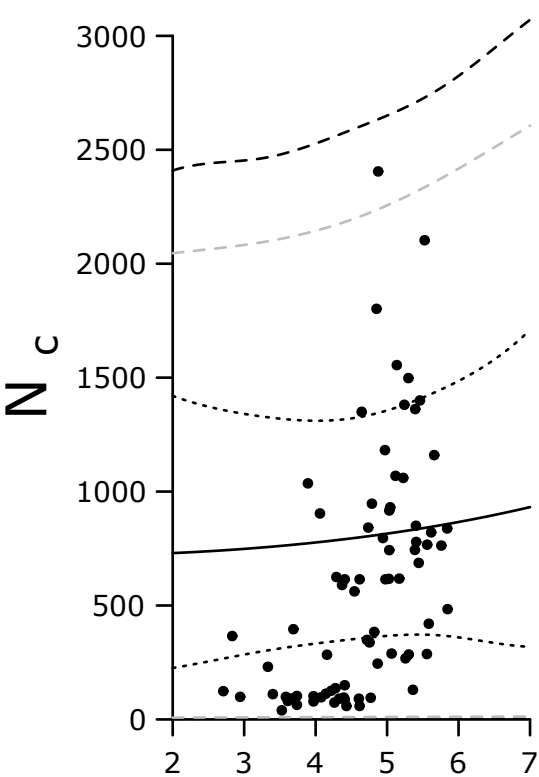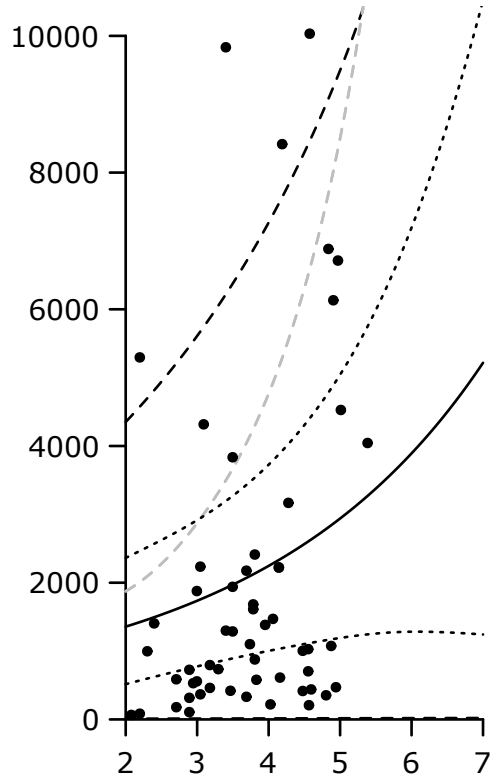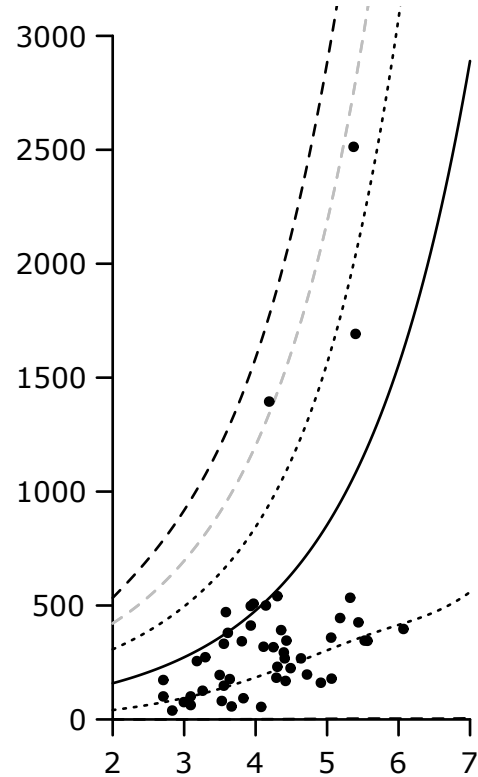

Lower CI  $\ln(N_b)$

Supplement: Supplementary file 1 [file EVA-10-935-s001.pdf]
