# Supplementary material for: A critical assessment of estimating census population size from genetic population size (or vice versa) in three fishes
Source: Evol Appl. 2017 Jul 4;10(9):935–45. doi: 10.1111/eva.12496 (PMC5680432; doi:10.1111/eva.12496)
Supplement: Supplementary file 2 [file EVA-10-935-s002.docx]

Table S1: Sample size (S), number of marked individuals (m), proportion of marked individuals during recapture in % (M), effective number of breeders calculated from LDNe (N_b_), adult census population sizes (N), and Nb/N ratios for Atlantic salmon populations coexisting within two Cape Race streams: Upper Ouananiche Beck (UO) and Watern (WN). See Bernos et al (submitted) for methods and complete analysis.

|  |  |  |  |  |  |  |  |
| --- | --- | --- | --- | --- | --- | --- | --- |
| **Cohort** | ***S*** | ***m*** | ***M*** | ***N_b_*_(LD)_** | **N** | ***N_b(LD)_/N*** | ***N_b(Sib)_/N*** |
| **UO11** | 51 | NA | NA | 100 (67-180) | NA | NA | NA |
| **UO12** | 29 | 113 | 39 | 23 (17-33) | 366 (295-475) | 0.06 | 0.10 |
| **UO13** | 44 | 49 | 29 | 35 (28-45) | 220 (274-115) | 0.16 | 0.25 |
| **UO14** | 34 | 82 | 18 | 57 (30-235) | 440 (336-612) | 0.13 | 0.10 |
| **WN12** | 48 | NA | NA | 55 (40-81) | NA | 0.14 | 0.12 |
| **WN13** | 33 | 43 | 10 | 80 (44-273) | 405 (240-931) | 0.20 | 0.09 |
| **WN14** | 54 | 27 | 22 | 62 (42-104) | 111 (67-345) | 0.56 | 0.50 |
