# Supplementary material for: A critical assessment of estimating census population size from genetic population size (or vice versa) in three fishes
Source: Evol Appl. 2017 Jul 4;10(9):935–45. doi: 10.1111/eva.12496 (PMC5680432; doi:10.1111/eva.12496)
Supplement: Supplementary file 3 [file EVA-10-935-s003.docx]

Table S1: Point estimates and 95% credible intervals (from posterior distribution) for heteroscedastic residual variance in a model predicting N_c_ from N_b_ at a gradient of population sizes representative of N_b_ estimates contained within the dataset. This model assumed decreasing residual variance with increasing N_b_ (term = “(idh(species:sqrt(1/ln(N_b_))):units)”).

| Species | N_b_ = 20 | N_b_ = 50 | N_b_ = 100 | N_b_ = 300 | N_b_ = 600 |
| --- | --- | --- | --- | --- | --- |
| Chinook salmon | 0.271 (0.114, 0.612) | 0.234 (0.113, 0.545) | 0.239 (0.097, 0.509) | 0.198 (0.083, 0.483) | 0.214 (0.080, 0.478) |
| Atlantic salmon | 0.092 (0.046, 0.187) | 0.078 (0.046, 0.151) | 0.074 (0.046, 0.137) | 0.074 (0.042, 0.124) | 0.068 (0.038, 0.119) |
| Brook trout | 0.160 (0.072, 0.354) | 0.157 (0.071, 0.304) | 0.121 (0.069, 0.286) | 0.126 (0.060, 0.267) | 0.121 (0.058, 0.265) |

Table S2: Point estimates and 95% credible intervals (from posterior distribution) for heteroscedastic residual variance (term = “(idh(species:sqrt(ln(N_x_))):units)”,) in a model predicting N_c_ from N_b_ at a gradient of population sizes representative of N_b_ estimates contained within the dataset. This model assumed increasing residual variance with increasing N_b_ (term = “(idh(species:sqrt(1/ln(N_b_))):units)”).

| Species | N_b_ = 20 | N_b_ = 50 | N_b_ = 100 | N_b_ = 300 | N_b_ = 600 |
| --- | --- | --- | --- | --- | --- |
| Chinook salmon | 0.208 (0.089, 0.470) | 0.257 (0.111, 0.525) | 0.266 (0.128, 0.584) | 0.287 (0.134, 0.676) | 0.269 (0.135, 0.738) |
| Atlantic salmon | 0.051 (0.028, 0.108) | 0.060 (0.036, 0.116) | 0.074 (0.041, 0.123) | 0.078 (0.046, 0.137) | 0.083 (0.051, 0.151) |
| Brook trout | 0.113 (0.051, 0.252) | 0.124 (0.062, 0.280) | 0.149 (0.072, 0.310) | 0.163 (0.081, 0.361) | 0.167 (0.083, 0.391) |

Table S3: Point estimates and 95% credible intervals (from posterior distribution) for heteroscedastic residual variance (term = “(idh(species:sqrt(1/ln(N_x_))):units)”) in a model predicting N_b_ from N_c_ at a gradient of population sizes representative of N_c_ estimates contained within the dataset. This model assumed decreasing residual variance with increasing N_c_ (term = “(idh(species:sqrt(1/ln(N_c_))):units)”).

| Species | N_c_ = 50 | N_c_ = 100 | N_c_ = 500 | N_c_ = 1 000 | N_c_ = 10 000 |
| --- | --- | --- | --- | --- | --- |
| Chinook salmon | 0.335 (0.154, 0.588) | 0.301 (0.151, 0.535) | 0.265 (0.133, 0.463) | 0.242 (0.124, 0.450) | 0.185 (0.097, 0.430) |
| Atlantic salmon | 0.166 (0.114, 0.366) | 0.162 (0.111, 0.319) | 0.165 (0.103, 0.269) | 0.142 (0.094, 0.257) | 0.118 (0.062, 0.243) |
| Brook trout | 0.119 (0.058, 0.296) | 0.116 (0.058, 0.264) | 0.110 (0.057, 0.225) | 0.109 (0.055, 0.218) | 0.092 (0.049, 0.206) |

Table S4: Point estimates and 95% credible intervals (from posterior distribution) for heteroscedastic residual variance (term = “(idh(species:sqrt(ln(N_x_))):units)”) in a model predicting N_b_ from N_c_ at a gradient of population sizes representative of N_c_ estimates contained within the dataset. This model assumed increasing residual variance with increasing N_c_ (term = “(idh(species:sqrt(1/ln(Nc))):units)”).

| Species | N_c_ = 50 | N_c_ = 100 | N_c_ = 500 | N_c_ = 1 000 | N_c_ = 10 000 |
| --- | --- | --- | --- | --- | --- |
| Chinook salmon | 0.203 (0.117, 0.421) | 0.259 (0.132, 0.444) | 0.285 (0.162, 0.528) | 0.308 (0.171, 0.572) | 0.354 (0.185, 0.731) |
| Atlantic salmon | 0.123 (0.073, 0.235) | 0.129 (0.088, 0.248) | 0.168 (0.108, 0.282) | 0.176 (0.110, 0.299) | 0.210 (0.118, 0.373) |
| Brook trout | 0.075 (0.037, 0.175) | 0.085, 0.042, 0.183) | 0.113 (0.056, 0.217) | 0.118 (0.055, 0.227) | 0.137 (0.065, 0.280) |
