# Supplementary material for: A critical assessment of estimating census population size from genetic population size (or vice versa) in three fishes
Source: Evol Appl. 2017 Jul 4;10(9):935–45. doi: 10.1111/eva.12496 (PMC5680432; doi:10.1111/eva.12496)
Supplement: Supplementary file 4 [file EVA-10-935-s004.docx]

Table 1: Published studies examining N_b_/N_c_ relationships in species for which limited data are available. Note that some studies did not use LDNe to estimate N_b_.

| Authors | Year | Species | Number of Populations | Total usable N_b_/N_c_ estimates |
| --- | --- | --- | --- | --- |
| Scribner et al | 1997 | *Bufo bufo* | 3 | 3 |
| Ardren and Kapuscinski | 2003 | *Oncorhynchus mykiss* | 1 | 3 |
| Brede and BeeBee | 2006 | *Rana temporaria* | 2 | 2 |
| Schmeller and Merila | 2007 | *Rana temporaria* | 2 | 2 |
| Brede and BeeBee | 2006 | *Bufo bufo* | 2 | 2 |
| BeeBee | 2009 | *Bufo calamita* | 6 | 6 |
| Ficetola et al | 2010 | *Rana latastei* | 9 | 9 |
| Hoehn et al | 2012 | *Oedura reticulata* | 4 | 7 |
| Christie et al | 2012 | *Oncorhynchus mykiss* | 1 | 11 |
